# Prospects in Connecting Genetic Variation to Variation in Fertility in Male Bees

**DOI:** 10.3390/genes12081251

**Published:** 2021-08-16

**Authors:** Garett P. Slater, Nicholas M. A. Smith, Brock A. Harpur

**Affiliations:** 1 Department of Entomology, Purdue University, 901 W State St., West Lafayette, IN 47907, USA; Slater20@purdue.edu; 2 School of Biological Sciences, The University of Queensland, Saint Lucia, QLD 4072, Australia; nick.smith@uq.edu.au

**Keywords:** honey bee, spermatogenesis, male fertility

## Abstract

Bees are economically and ecologically important pollinating species. Managed and native bee species face increasing pressures from human-created stressors such as habitat loss, pesticide use, and introduced pathogens. There has been increasing attention towards how each of these factors impacts fertility, especially sperm production and maintenance in males. Here, we turn our attention towards another important factor impacting phenotypic variation: genetics. Using honey bees as a model, we explore the current understanding of how genetic variation within and between populations contributes to variation in sperm production, sperm maintenance, and insemination success among males. We conclude with perspectives and future directions in the study of male fertility in honey bees and non-*Apis* pollinators more broadly, which still remain largely understudied.

## 1. Introduction

Bees (*Anthophila*) are economically and ecologically important insects that, mostly, act as pollinators. They improve the production of 87 of the leading global food products and provide over USD 200 billion worth of ecosystem services [1]. In the United States alone, there are over 4000 native and non-native bee species [2]. The Western honey bee (*Apis mellifera*; henceforth honey bee) is the most well-recognized pollinator in North America [3], and provides a substantial portion of pollination services on the continent. They are non-native to the Americas and have been continually imported for at least 400 years [4]. Populations in the United States originate from at least nine different genetically and phenotypically different population groups (subspecies) [4]. Honey bees are largely found in commercial operations where they are managed for honey production and pollination. Other managed species such as the solitary leaf cutter bees (*Megachile rotundata*) [5] and social bumble bees (*Bombus* spp.) [6] also provide pollination services to orchards and greenhouses [7]. Wild, native bees such as solitary squash bees (*Eucera (Peponpis) pruinosa*) and other bumble bees are also critically important to crop pollination and to ecosystem health [8]. Declines or losses of both native and non-native bee species can impact agricultural production and ecosystem function [9].

The causes of pollinator decline and loss are multifactorial [10]: habitat loss, pesticide use, and pathogen pressure all account for pollinator losses to date and each contributes to pollinator decline in different ways. For example, by reducing the area available for species to nest, destroying or contaminating food, and outright killing populations [10]. In honey bees, infertility is a major factor in colony decline. As many as 50% of queens die within 6 months [11,12] and much of this mortality is directly linked to sperm quality [11,13,14]. Despite this, there is a dearth of research on the mechanisms through which variation in male fertility arises. We have a detailed and growing understanding of how variation in male bee fertility is influenced by environmental factors [15,16,17]; however, we have much less understanding of the genetic and mechanistic bases underpinning how variation in male fertility arises. This is a major gap in both our understanding of bee biology and in our ability to effectively manage and conserve declining bee populations. In this review, we focus on honey bee male fertility as a model to understand the genetic factors shaping sperm traits. We conclude with perspectives and future directions in the study of male fertility in honey bees and non-*Apis* pollinators more broadly, which still remain largely understudied.

## 2. An Overview of Male Reproductive Biology in Honey Bees

In order to understand how variation in male fertility arises, it is first important to understand honey bee mating biology. Honey bees are eusocial insects, meaning they have a reproductive division of labor, overlapping adult generations, and cooperative brood care [18]. They also live in large colonies consisting of thousands of non-reproductive workers that perform most colony tasks and a single reproductive queen that specializes in egg laying. Queens lay either fertilized (diploid) or unfertilized (haploid) eggs. Diploid eggs typically develop into either queens or workers (both female), depending on the diet provided to them by workers during development [19]. Haploid eggs develop into drones (male honey bees).

Queens begin mating flights 5–6 days after emergence and make 1–5 mating flights over the course of a week [20]. During these mating flights, queens mate with an average of 12 males [21] and store their sperm in a specialized organ called the spermatheca. Post-mating, the queen undergoes both morphometrical and physiological changes to specialize in egg laying [22]. Most notably, the queen’s ovaries will increase in size to create 150–180 egg-producing ovarioles [23]. This allows queens to produce thousands of fertilized eggs per day. Egg production is not a limiting resource for queens, but they are limited in the number of sperm they can store. Queens only store 3–8 million sperm cells [24,25], which will be used during their 1–2 year lifespan. The non-reproductive workers in a colony will replace the queen when the production of viable offspring from fertilized eggs declines. Thus, a queen’s longevity and fertility is dependent on the ability to mate and store sperm.

**Figure 1:**
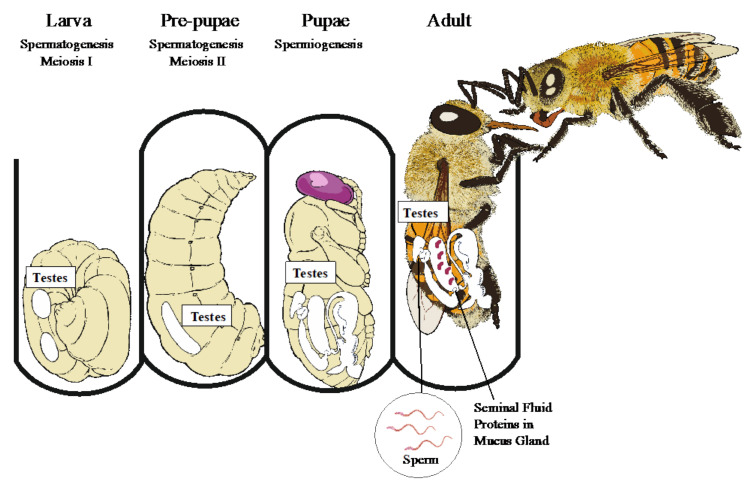
Sperm production in honey bee drones during development. Spermatogenesis, the production of sperm, occurs during late larval and pre-pupae development. Spermiogenesis, sperm differentiation, occurs during pupation. Once honey bee drones emerge as adults, sperm production is already complete. However, drones will produce seminal fluids in the mucus glands during early adult emergence. Photo credit: Amy Geffre.

**Figure 2:**
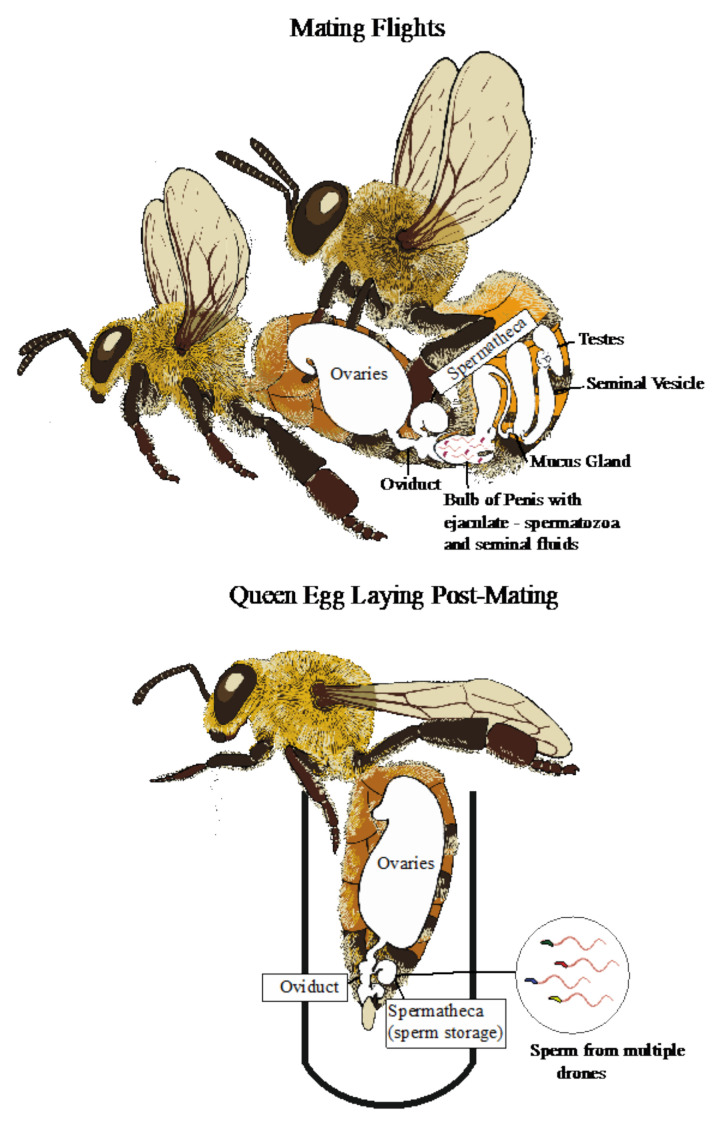
During insemination, drones transfer both spermatozoa and seminal fluids into the queen’s oviduct, which will eventually become stored in the spermatheca. Once stored, queens will use this sperm until death. Photo credit: Amy Geffre.

When drones eclose, they will have produced all of the spermatozoa they will ever produce in their lifetime [26] (Figure 1). At eclosion, the sperm is stored in the testes, but will migrate into the seminal vesicles when the drone becomes sexually mature. This begins immediately after drones emerge, but the sperm does not fully migrate until two weeks after emergence [27,28]. If sperm migration is slow, then drones will be immature and cannot fertilize a female [29]. Drones will begin orientation and mating flights around 8 days old [30], even though all their sperm has not fully migrated. Little is known about the exact nature of mating because it occurs in the air during flight; however, drones often congregate along their flight paths in Drone Congregation Areas (DCAs). From here, any passing queens will be chased by the drones who compete for access to mate [31,32,33]. Drones will form ‘comets’ behind the unmated queen until one of the drones reaches, mounts, and inseminates the queen (Figure 2). A successful drone, upon mounting, will evert the endophallus through the queen’s sting chamber and into the oviduct. The tip of the everted endophallus consists of cervical plates, coagulating mucus proteins from the mucus gland within the bulb tube, and a sticky orange lipid secretion from the cornual gland [34]. The mucus-filled endophallus forms a strong connection between the drone and the queen, and the sticky orange secretion further strengthens this connection. Mating lasts a few seconds [35,36] and propels sperm and seminal fluids into the oviduct (Figure 2). Following ejaculation, the endophallus breaks off and the male dies.

In the oviduct, sperm is tightly clumped [37] and unable to swim to the spermatheca. To facilitate the migration from the oviduct to spermatheca, the queen performs muscle contractions [37]. This action causes sperm loss by the queen as only about 2.5% of the sperm produced by each drone makes it into the spermatheca [37]. It is at the spermatheca that the sperm tail unfolds. Honey bee spermatozoa have the longest tails of any bee analyzed to date [38] and it has been suggested that tail length may function in sperm competition [37] and/or storage to increase the likelihood of fertilization [39]. Successful fertilization occurs when a queen secretes sperm from the spermatheca to an egg passing through the oviduct. The number of sperm secreted varies over the course of the queen’s lifespan but a queen averages two sperm per fertilized egg [40]. While there is no direct evidence of sperm competition in honey bees to date, it has been suggested that sperm may compete at this stage to fertilize eggs [37]. Queens store the sperm they have collected from drones in the spermatheca for the remainder of their lives (up to 5 years) [23]. By the time a queen is replaced by the colony, she will have used only about 1% of the stored sperm to fertilize eggs [37].

Drones produce sperm only during larval and pupal development, so these early stages are essential for sperm production and quality. Environmental stress during development can influence a drone’s reproductive quality [16]. For example, drones raised in resource-limited colonies produce less concentrated sperm [41]. Spermatogenesis begins during embryogenesis with the formation of testes. The testes are composed of 150 testiolar tubules [42] and these testiolar tubules are packed with primary spermatogonia or undifferentiated germ cells [26]. During the final stage of larval development (fifth instar), the primary spermatogonia undergo four rounds of mitosis to form 16 secondary spermatogonia. The secondary spermatogonia are then encapsulated by somatic insect-specific cyst cells that are rich in mitochondria, glycogen, and nutrient-secreting cells [43]. Within these cyst cells, secondary spermatogonia undergo a series of synchronous mitotic divisions before entering spermatogenesis as primary spermatocytes. Spermatogenesis involves two rounds of meiotic cell division from late larval to pre-pupal development. During meiosis I, the primary spermatocytes begin to enter meiotic cell division, but the nuclear envelope fails to break down. This results in an aborted cell division [26,44]. In the bees, aborted meiosis seems to be a honey-bee-specific trait and results in secondary spermatocytes that are the direct product of the primary spermatocytes [42]. Once drones enter pre-pupation, the secondary spermatocytes undergo a successful second meiotic division and this produces spermatids of two different sizes [42]. It is unknown whether both spermatids develop into spermatozoa or whether the smaller spermatid disintegrates. After meiosis, each cyst contains an average of 202.8 spermatids [43].

Even though spermatozoa are produced only once during development, the total ejaculate is fully produced once drones emerge as adults. The complete ejaculate also includes seminal fluids that are produced in accessory glands (the mucus glands and ejaculatory duct [45]) and other proteins, carbohydrates [46], metal ions [46], and lipids [47]. The seminal fluids interact with sperm to improve quality, extend storage, promote fertilization, and reduce competition from other males [48,49]. Seminal fluid production increases immediately after emergence and maximizes after drones are 5–6 days old [45]. However, this could depend on the colony environment as workers feed and care for drones until they begin mating flights. Post-mating, the entire ejaculate, including seminal fluids and spermatozoa, is passed to the queen when inseminated (Figure 2).

## 3. Sperm Traits Vary Considerably among Honey Bee Populations

Sperm is central to a drone’s reproductive ability. Any variation in sperm quality or quantity can directly impact its ability to reproduce. There have been several studies exploring how environmental variation contributes to variation in sperm phenotypes and fertility: nutrition, agrochemical treatment, and temperature during larval and adult life stages. Each of these factors contribute to a drone’s ability to produce and maintain sperm [13,50,51,52,53,54]. One understudied and overlooked source of variation is genetic variation. Common garden experiments (studies comparing subspecies or breeding lines in replicate environments) are one method to determine potential genetic contributions to phenotypes. Common garden experiments are possible in honey bees, especially in their native ranges where genetically distinct populations co-exist. Unfortunately, there are few such studies to date on any sperm-related phenotypes (Table 1). However, the few studies to date suggest that there may be a strong genetic component to drone reproductive variance. *A. m. carnica* drones have larger mucus glands, testes, and seminal vesicles compared to *A. m. jemenetica* and they also have significantly more sperm in their ejaculate [55]. Similarly, *A. m. siciliana* and *A. m. ligustica* have significant differences in sperm concentration and sperm longevity [56]. Outside their native range, comparisons between imported European-sourced *Apis m.* and African-sourced *A. m. scutellata* show that sperm traits vary among subspecies [57] (Table 1). Studies on breeding lines have found significant variation in reproductive senescence [58], sperm traits [38,59,60], seminal fluids [61], morphology [55], flight [62], and response to stressors [63]. Collectively, these studies suggest that genetic variation in sperm traits exist, but this is a major gap in our understanding. There has yet to be a rigorous examination into how much of this variation is due to genetic differences and there are currently no estimates of heritability for any fertility-associated traits in honey bees.

## 4. Connecting Genetic Variation to Phenotypic Variation

With only a few studies exploring genetic differences in reproductive traits, it is difficult to pin down precisely what mechanistic and evolutionary processes have led to this variance. However, the biology of sperm production, sperm maintenance, drone reproductive morphology, and sperm storage has received significant research attention and provides a useful leaping-off point to describe points where variation could arise, venturing outside of honey bees to other non-model bees (e.g., bumble bees) or to the model insect *Drosophila*. The following sections will explore which traits are important for a drone’s reproductive health and how variation may arise.

## 5. Importance and Determinants of Sperm Concentration and Quality to Drones

The concentration of sperm presented on the endophallus is predictive of a drone’s reproductive success [66,67]. More concentrated sperm increase a drone’s probability of getting sperm pumped through the oviducts into the spermatheca, which are then used for fertilization. Since sperm competition is likely non-existent or limited in honey bees [37], drones producing more sperm have a greater probability of fertilizing eggs. For example, sperm inseminated at higher quantities had greater paternal frequencies than sperm at lower quantities [66,67]. Additional studies found smaller drones had lower reproductive success, and they attributed this to lower sperm quantity [68]. A second but under-investigated benefit is sperm cooperation. Sperm tend to clump within the oviduct and related sperm likely clump together. This may allow sperm to exhibit group behavior and exchange beneficial materials to mitigate negative competition from unrelated sperm, improve long-term storage, and increase fertilization probability. While sperm cooperation has not been tested in honey bees, it has been shown in other insects [69]. Variation in sperm quality can be the result of variation in tubule number, in the quality of spermatogenesis, in sperm migration from the testes, in the success of ejaculation and migration from the oviduct to the spermatheca, and of course in how well that sperm can be stored.

### 5.1. Tubule Number

Spermatogenesis (sperm production) occurs in the testiolar tubule and morphological or physiological variation in these tubules is expected to change sperm production [70]. For example, bumble bees have shorter (and likely fewer) testiolar tubules and they produce only 3 million sperm per male [71] whereas honey bees have over 200 long testiolar tubules [42] and produce an average of 7 million sperm per drone [50,51]. It is unknown how much variation there is in testiolar number among honey bees, but newly emerged drones do vary in testis size [55,72], a trait highly correlated with testiolar tubule number. Comparing isolated populations, the subspecies *A. m. jemenetica* testes had a volume of 20.76 mm^3^ compared to 30.43 mm^3^ in *A. m. carnica* [55].

### 5.2. Spermatogenesis Quality

Spermatogenesis in the testiolar tubules occurs in late-larval to pre-pupal stages. Any variation in spermatogenesis impacts the number of sperm produced. Compared to other Hymenoptera, honey bees have all the core meiotic genes, except for three genes involved with meiotic recombination: *DMC1, RAD51C*, and *RECQ3* [73]. These core meiotic genes are involved with cell cycle control, chromosomal structure maintenance, and meiotic recombination during spermatogenesis. Honey bees also have unique abortive meiosis I and anomalous meiosis II [44], which appear to be determined by the genes *bol* and *crl* [74]. The gene *boule* (*bol)* is a member of the Deleted in Azoospermia (DAZ) gene family, which has known functions in meiosis and sperm differentiation [75]. In sawflies (Hymenopteran, suborder *Symphyta*), three alternatively spliced transcripts of *bol*, *Ar bol, Ar bol 2*, and *Ar cdc25*, are highly expressed during spermatogenesis. RNA interference (RNAi) knockouts of these three transcripts arrests spermatogenesis and no mature sperm have been identified post-emergence [76]. These genes are likely to be associated with the G_2_/M transition and sperm differentiation stages of spermatogenesis. The gene *courtless (crl)* is also involved with meiosis in honey bees. In *Drosophila*, *crl* mutants do not undergo meiosis during spermatogenesis and these mutants produce abnormal sperm [77].

### 5.3. Migration from the Testes

Once drones emerge as adults, sperm must migrate from the testes to the seminal vesicles (Figure 1). This migration delays sexual maturity [29,78,79]. Therefore, if sperm migration rates vary then sperm concentration is determined post-emergence. Sperm migration does vary among drones [80] and it is likely to have a genetic component. Drones from distinct breeding populations differ in the rate of sperm migration into the seminal vesicles [58]. Drones also differ in the ability to copulate [60] which suggests that there is slower sperm migration. The mechanisms underlying this variation are unknown.

### 5.4. Ejaculation and Sperm Movement

Once sperm enters a queen’s oviduct, it moves to a storage organ known as the spermatheca (Figure 2). Ejaculated sperm possesses the biochemical machinery needed to perform aerobic metabolism [81]. In honey bees, sperm motility and flagellar movement is energetically exhaustive [82] and requires ATP-dependent energy production for competition between drones and storage [83]. Once the honey bee sperm is stored in the spermatheca, it starts to produce ATP by acidifying glycolytic metabolism using anaerobic metabolisms rather than aerobic metabolism [81]. The queen is likely to maintain an oxygen-depleted spermatheca that functions to reduce energetic costs of sperm during storage, reduce ROS activity, and allows stored sperm to undergo anaerobic metabolism. This has been confirmed by the upregulated levels of the enzyme metabolizing GA3P [81] and of glycolytic proteins during storage [84]. Additionally, when the GA3P enzyme degrades during long-term storage, sperm quality seems to deplete [83].

To further understand the molecular mechanisms of this aerobic metabolism, *Drosophila* provide a useful model. *Drosophila* proteomic analysis found sperm proteins are involved with metabolic and respiratory pathways, and these proteins are conserved across insect species [85,86,87,88]. These metabolic pathways are enriched with metabolic processes, including carbon metabolism (e.g., pyruvate and butyrate metabolism, the TCA cycle, and oxidative phosphorylation), and the metabolism of several classes of amino acids [88]. The most transcriptionally abundant proteins in *Drosophila* testis and sperm are Sperm-Leucylaminopeptidase, S-Lap1 and loopin [86,87,89], which are structural components of mitochondrial paracrystalline material. RNAi knockout of this gene family demonstrates that it is essential for male fertility [90]. This could explain its rapid expansion in *Drosophila* [91]. In addition, several respiratory pathways were found in *Drosophila* sperm, including several cytochromes (CoVa, Cyt-c-d) [87]. This supports the role of aerobic metabolism in insect sperm to produce energy for motility and fertilization. *Drosophila* sperm also contains several proteins associated with sperm motility [85,86,87,92]. In addition to metabolism, the *Drosophila* genome contains several genes associated with sperm movement and motility. These motility proteins can be categorized as structural (*αtub84B*, *βTub85D*, *βTub56D, Act5C, Gas8*), developmental (e.g., *blw*, *dj*, *heph*, *Hsp83*, *jar* and *ox)*, electron transport (*mtacp1)*, and sperm individualization proteins. While these genes have been found in *Drosophila*, they may have an important role in honey bee sperm movement and metabolism.

### 5.5. Sperm Storage by the Queen

Queens produces spermathecal fluid to maximize the long-term storage of sperm. This fluid ensures an appropriate environment for the long-term viability of sperm [93], including eliminating sperm competition [49]. It differs from seminal fluids [84] and contains proteins associated with energy metabolism and antioxidant defense. This is similar to other insects that have spermathecal fluids that contain proteins associated with metabolic function (e.g., Yolk Protein-1, vitellogenin, cytochrome P450, lipase-3) [94]. The honey bee spermatheca provides proteins involved with anaerobic metabolism and the glycolytic pathway [84], which are nonexistent proteins in seminal fluids. When the sperm enters the spermatheca, its source of sugar is altered to the fructose provided by the spermatheca. This shift towards fructose allows sperm to use the glycolytic pathway and reduce their metabolic rate. Queens must store sperm for 1–2 years. Therefore, they also produce antioxidants to improve storage. Mated compared to unmated queens had higher upregulation of cytochrome p450 and the antioxidant proteins catalase, TXN2 (Thioredoxin, mitochondrial precursor), TXNRD1 (Thioredoxin reductase 1), GSTD1 (Glutathione S-transferase), and SOD1 (Superoxide dismutase 1) [95,96,97,98,99]. Thioredoxin has been confirmed multiple times and it plays an important role in the protection of sperm during storage. It also exposes sperm chromatin during fertilization [100]. Only two of the seven genes in the thioredoxin family in the honey bee genome appear to be expressed but they are likely to have an important role in sperm protection [101]. Mated queens also have more metabolites associated with glycophospholipids [102]. These abundant lipids protect sperm against peroxidation via superoxide dismutase and the glutathione peroxidase/reductase system [103].

## 6. Importance of Sperm Morphology to Drone Fertility

Drones have the longest and largest sperm among closely related bees [38,104]. This has been attributed to the evolution of polyandry (multiple mating) in honey bees and the advent of sperm competition [104]. However, sperm competition is minimized in honey bees because sperm clumping eliminates the drone sperm’s ability to swim within the queen’s oviduct [105,106]. Another explanation is that longer and bigger sperm live longer during storage. Recent evidence in *Drosophila* found male sperm length increased when sperm storage organ length is selected upon [107]. In bees, comparative work found a strong correlation between sperm length and sperm storage organ [108,109,110,111,112]. Longer sperm may be better suited for long-term storage due to increased longevity, energy production, and sperm displacement within the sperm storage organ. Considering queens live on average 1–2 years, longer-lived sperm have a greater probability to fertilize eggs, so this could cause sperm length and morphology to be highly selected upon.

### Spermiogenesis

Spermiogenesis is the elongation and differentiation of spermatids to spermatozoa and this process determines sperm morphology. Honey bee spermiogenesis is almost identical to *Drosophila* [42], so the molecular mechanisms are likely to be similar. One difference is the use of the storage protein hexamerin during spermiogenesis. Hexamerin is produced by the larval fat body, broken up, and used as an amino acid resource during metamorphosis [113]. A protein subunit of hexamerin, HEX 70a, is found in drone testes and is likely involved with spermiogenesis. HEX70a is found in the nucleus of non-proliferated spermatids and individualized spermatozoa [114]. Thus, this protein may be associated with spermatid differentiation.

## 7. Importance of Seminal Fluids to Drone Fertility

Adult honey bees males have a fixed number of sperm, so it is critical for males to produce high-quality ejaculates that are viable during copulation, sperm transfer, and sperm storage [115]. In honey bees, seminal fluids have a wide range of functions, which have been reviewed previously [15]. Seminal fluids mainly function to protect sperm. For example, seminal fluids include proteins involved with detoxification, immunity, and oxidative stress [61]. Seminal fluids also interact with the queen to increase fertilization by increasing egg laying rate, improving spermatheca sperm storage, and altering female receptivity to sperm [15]. Lastly, seminal fluids are involved with sperm competition. A study found sperm viability is reduced when exposed to genetically dissimilar seminal fluids [116]. Because sperm clumping reduces sperm swimming [37], seminal fluids may be used to gain a fitness advantage over other sperm.

There are as many as 260 seminal fluid proteins identified in honey bees [61], many of which are likely sperm-protective [15]. Honey bee males produce at least fifteen proteins linked to insect immunity pathways, such as the Toll pathway, immune deficiency (IMD), c-Jun N- terminal kinases (JNK) signaling, and Janus kinase/signal transducers and activators of transcription (JAK/STAT). The abundance of insect immunity proteins indicates that seminal fluids have an important protective role against foreign material, such as Gram-positive bacteria, Gram-negative bacteria, fungi, and yeast. Two well-known proteins expressed include two chitinases and peritrophin-1 which have known functions in the Toll pathway. These proteins bind and break down the chitin cell walls of parasitic fungi. Another protein includes peptidyl-prolyl cis−trans isomerase, which activates and modifies antimicrobial protein (AMP) release into the seminal fluid. Seminal fluid proteins also include proteins with known defenses against oxidative stress, such as superoxide dismutase 2 [48]. There are additional proteins in the seminal fluids and these have several other functions, such as supporting sperm metabolism [48], increasing female egg laying [15], female vision [117], and protection against foreign sperm [49].

Seminal fluids are much more studied in other insects compared to honey bees. Insect seminal fluids are involved in antimicrobial activity [118,119], sperm maintenance and storage [49,115,120,121], receptivity to remating [122,123,124,125,126,127], and physiological and behavior changes to females [128,129,130]. There are several classes of proteins with conserved functions among insects. These proteins include proteolysis (e.g., trypsins, a zinc carboxypeptidase, a metalloprotease, a serine protease inhibitor (serpin)) [131,132,133,134] that regulate liquefaction and maturation of semen in the female reproductive tract [135,136,137,138,139] and intercellular proteins (e.g., ATPases, dipeptidyl peptidase, γ glutamyl transpeptidase, glutathione S-transferase, angiotensin converting enzyme) that regulate accessory gland secretion [132,140,141]. Many seminal fluids are species-specific [123] with a wide range of functions.

## 8. Future Directions

We are beginning to develop a molecular understanding into how variation in components of honey bee sperm arise. However, compared to *Drosophila*, there is still a substantial gap in our understanding of how drones produce and maintain their ejaculates throughout their lifespan, how genetic differences contribute to variation in their ability to do this, and even in how we determine which traits are critical to determining a drone’s fitness.

### 8.1. On the Need to Connect Sperm Quality to Fertility in Bees

We focus our review on understanding how variation in sperm traits may arise, but one key question remains in all work to date, and that is understanding the connection between sperm trait variation and fertility. Currently, we do not know which traits are the best predictors of sperm quality. Most studies measuring sperm quality use sperm viability as measured by dual fluorescent staining as a proxy [80,142]. Other proxies could provide a more precise measurement of sperm quality. For example, mitochondrial activity, acrosome reaction, DNA fragmentation, and sperm motility could be used [143]. Mitochondrial activity measures sperm metabolism and energetics. This trait is important during aerobic metabolism when the sperm is in the oviduct because sperm movement to storage is energetically exhaustive. Acrosome reaction measures the sperm’s ability to fertilize eggs by replicating fertilization. The sperm may be viable but has a reduced capacity to fertilize eggs [144]. DNA fragmentation detects damaged DNA in the sperm [143]. DNA damage in sperm can occur for several reasons, such as environmental variation. Sperm motility measures sperm movement and velocity and is associated with sperm quality. These alternative proxies may provide a more accurate measurement of sperm quality and performance.

Studies also vary in the methods used to extract and quantify sperm [80,145,146]. Two primary methods have been employed. The first method forces drones to release sperm by stimulating mating. Drones evert their endophallus after pressure is applied to the abdomen or after being exposed to chloroform. At this stage, sperm can be collected at the tip using a syringe [146]. This method measures the sperm drones would use during mating. The second method measures sperm in the seminal vesicles [80]. This method requires dissecting the seminal vesicles and tearing the tissue in a buffer to force sperm release. This method measures the sperm migrating from the testes to the seminal vesicles, but it does not accurately measure the sperm a drone would use during mating. While these two methods have been used interchangeably, they answer different questions and future studies should consider which method is more appropriate for their study. In addition, studies should control for changes in sperm due to age, nutrition, and other environmental stressors. This can be achieved by measuring sperm in the testes immediately after drone adults emerge. This approach measures the potential sperm drones could use during mating.

Future work should connect sperm phenotypic variation to fertility and fitness. As mentioned above, several sperm traits likely provide fitness benefits for males. For example, highly concentrated sperm is likely to increase a drone’s probability of fertilization [66,67] and longer sperm may be better suited for long-term storage. Despite this, we still do not know which sperm traits vary and how they impact drone reproductive success. This inhibits our ability to effectively manage honey bee populations because we do not know which drone fertility traits to select. As we learn more about drone trait variation, targeted breeding programs can be established.

### 8.2. Identifying Candidate Genes

Studies to date (Table 1) suggest a genetic component but substantially more work is needed to understand the degree to which genetics plays a role in drone fertility and potentially identify genetic factors contributing to male performance. Candidate genes can be identified by comparing the genes of populations or distinct breeding lines where sperm traits vary. This can be done by performing genome-wide association studies (GWAS) or producing genetic crosses from these populations. GWAS require phenotyping a large number of individuals with variation in the trait of interest, sequencing their DNA, and identifying genomic regions associated with the trait. GWAS can be expensive and time-consuming. However, it is a good method for non-honey-bee pollinators because genetic crosses are more difficult to perform. Genetic crosses require distinct breeding lines or populations that express variation in a trait. Hybrids are produced from these lines or populations and the candidate loci associated with the trait are identified by comparing the genes of the hybrids and the parent populations. Genetic crosses have been widely used in honey bees to identify candidate loci for several traits, such as grooming and worker reproduction [147]. Once variation is identified for sperm traits important for fertility, these methods can be used to identify candidate loci.

Measuring heritability is the first step to identifying candidate genes. Heritability can be measured using the crosses or by comparing haploid brothers in the GWAS. Heritability can be used to identify candidate loci and QTLs associated with bee male fertility traits. While heritability only provides information on the genetic influence on traits, it can determine the statistical power in gene-mapping studies [148]. In *Drosophila*, studies have found candidate genes associated with sperm storage [149,150], sperm morphology and motility [151], seminal fluids [152], and sperm viability [153,154]. Heritability allows us to detect the genetic architecture of the trait and determine which genes shape their variation and quality more easily.

The causes of pollinator declines are multifarious, but among the largest threats is male sub-fertility or infertility. Male quality has an important role for queen quality, colony performance, and overall stability. As male quality declines, genetic diversity declines and inbreeding increases. This depletes offspring viability and lowers queen fertility and lifespan. It also exacerbates population declines by making populations more susceptible to pests, pathogens, poor nutrition, and pesticides. While much attention has focused on female quality, male fertility is vital for bee populations. We know comparatively less about the role genetics plays in shaping male reproductive traits. This is a major gap in both our understanding of bee biology and in our ability to effectively manage and conserve declining populations.

## Figures and Tables

**Table 1:** Common garden experiments among honey bee subspecies that have specifically examined sperm- or fertility-associated traits in honey bee males. Drone characteristics among honey bee subspecies indicate that there is a genetic component to drone reproductive traits. NA = Not Available; sig = Significant Differences; nsig = No Significant Differences.

	Reproductive Tract Morphology					Sperm Morphology and Semen Traits				
Comparison	Location	Weight	Testis	Seminal Vesicle	Mucus Gland	Length	Count	Volume	Longevity	Citation
*Apis mellifera jemenitica* vs. *A. m. carnica*	Saudi Arabia	sig	sig	sig	sig	NA	sig	NA	NA	[55]
*A. m. caucasica* vs. *hybrid A. m. carnica x caucasica*	Poland	NA	NA	NA	NA	nsig	NA	NA	NA	[64]
*A. m. siciliana* vs. *A. m. ligustica*	Sicily	NA	NA	NA	NA	NA	sig	nsig	sig	[56]
*A. m. syriaca* vs. *A. m. ligustica*	Jordan	sig	NA	NA	NA	NA	sig	NA	NA	[65]
*A. m. scutellata x managed* vs. *managed*	Venezuela	sig	NA	nsig	nsig	NA	sig	NA	NA	[57]
